# Late-stage interventional trials reporting sleep-related outcomes in older adults with Parkinson’s disease: a ClinicalTrials.gov registry review

**DOI:** 10.1186/s12877-026-07089-3

**Published:** 2026-02-17

**Authors:** Fahad J. Alqahtani, Mohammed M. Aldurdunji

**Affiliations:** 1 Pharmaceutical Sciences Department, College of Pharmacy, Umm Al-Qura University, Makkah, Saudi Arabia; 2 Pharmaceutical Practices Department, College of Pharmacy, Umm Al-Qura University, Makkah, 21955 Saudi Arabia

**Keywords:** Parkinson’s disease, Sleep disturbances, Older adults, Frailty, Dopaminergic therapy, Cognitive-behavioral therapy for insomnia, Geriatric research

## Abstract

**Background:**

Sleep disturbances are among the most disabling non-motor symptoms in Parkinson’s disease (PD), particularly affecting older adults, in whom age-related vulnerability and multimorbidity may exacerbate sleep dysfunction. Despite their high prevalence, interventional evidence reporting sleep-related outcomes in PD remains incompletely characterized, and methodological heterogeneity limits the comparability of findings across studies. This registry-based descriptive review aimed to characterize the landscape of late-stage interventional trials reporting sleep-related outcomes in PD, with attention to study design, older-adult eligibility, and intervention types.

**Methods:**

A descriptive analysis of completed late-stage interventional studies registered on ClinicalTrials.gov was conducted. Late-stage was operationally defined as Phase III–IV interventional trials and explicitly linked open-label extension/rollover studies. Eligible studies permitted enrolment of adults aged ≥ 65 years with Parkinson’s disease and reported at least one sleep-related outcome. Extracted data included intervention type, study design, sleep outcome measures, and study duration.

**Results:**

Twenty-one trials met inclusion criteria. Most focused on dopaminergic or adjunctive pharmacologic interventions, including rotigotine, pramipexole, and levodopa–carbidopa intestinal gel. Reported sleep-related outcomes were primarily derived from subjective instruments such as the Parkinson’s Disease Sleep Scale (PDSS/PDSS-2) or SCOPA-Sleep, while objective assessments such as polysomnography or actigraphy were infrequently specified. No completed late-stage trials evaluating structured behavioral or circadian-oriented interventions, including cognitive-behavioral therapy for insomnia, exercise, or bright-light therapy, were identified. Eligibility criteria suggested that clinically vulnerable populations, including frail or cognitively impaired individuals, were likely underrepresented.

**Conclusions:**

Late- stage interventional evidence reporting sleep-related outcomes in PD remains predominantly pharmacologic, short-term, and methodologically heterogeneous. The absence of completed behavioral or multimodal trials and eligibility criteria that may limit inclusion of clinically vulnerable individuals highlight important evidence gaps. Future research should prioritize inclusive, geriatric-informed, and multimodal designs incorporating both subjective and objective sleep measures.

## Introduction

Sleep disturbances, including insomnia, excessive daytime sleepiness and rapid eye movement sleep behaviour disorder, are common and disabling non-motor features of Parkinson’s disease, affecting approximately 60 to 80% of people and impairing fatigue, cognition, falls and quality of life [1–5]. In older adults, who represent a substantial proportion of the Parkinson’s population, these problems are intensified by age-related vulnerability, multimorbidity and polypharmacy.

Distinct contributions from each disturbance have been described. Insomnia has been reported in 48 to 76% of individuals and may appear early, with progression linked to motor and non-motor deterioration [6–8]. Excessive daytime sleepiness has been attributed to nocturnal fragmentation, dopaminergic overstimulation and degeneration of arousal networks, and has been associated with worse function and higher caregiver strain [9–11]. Rapid eye movement sleep behaviour disorder has been observed in up to 50% of patients and has been linked to greater disease severity, autonomic dysfunction and cognitive decline, with occurrence before motor symptoms in some individuals [12–14].

A multifactorial neurobiological basis has been implicated, involving dopaminergic, serotonergic and hypothalamic systems. Nocturnal fragmentation and daytime somnolence have been associated with degeneration and treatment effects; dopaminergic therapy may relieve nocturnal akinesia, yet daytime sleep attacks and excessive sleepiness have been reported [8, 15–17]. Single-agent pharmacological therapy rarely normalises sleep, which supports multimodal strategies.

Evidence for treatment has remained mixed. Dopamine agonists such as pramipexole and rotigotine have been used widely, with reports of improvements in nocturnal symptoms accompanied by increased risk of excessive daytime sleepiness and sleep attacks [18–20]. Non-pharmacological options, including cognitive behavioural therapy for insomnia, structured exercise and bright-light therapy, have shown early signals of benefit, although adequately powered randomised trials explicitly designed for frail older adult populations remain scarce [21–23].

Methodological limitations have further constrained inference. Many Parkinson’s sleep studies have relied on subjective instruments such as the Parkinson’s Disease Sleep Scale, the Scales for Outcomes in Parkinson’s Disease–Sleep and the Pittsburgh Sleep Quality Index, while objective assessment with polysomnography or actigraphy has been infrequently specified [24, 25]. Older adults with frailty or cognitive impairment are likely to be under-represented because of restrictive eligibility, which limits generalisability to real-world geriatric practice [26, 27]. These gaps have underscored the need for inclusive, standardised and multimodal approaches to the management of sleep disturbance in Parkinson’s disease [28, 29].

The aim of this study was to conduct a registry-based descriptive review of completed late-stage interventional trials (Phase III and Phase IV) registered on ClinicalTrials.gov that reported sleep-related outcomes in Parkinson’s disease and permitted enrolment of adults aged 65 years or older. Specifically, the review sought to identify and characterize eligible trials, summarize intervention types, study designs, and sleep outcome measures, and examine age-related eligibility criteria relevant to older adults, in order to highlight methodological patterns, evidence gaps, and priorities for geriatric-focused research and clinical practice.

## Methods

### Study design and data source

This study was conducted as a registry-based descriptive review of completed interventional trials in Parkinson’s disease (PD). Data were obtained from the publicly accessible ClinicalTrials.gov database, which provides structured information on trial design, eligibility criteria, interventions, and reported outcomes. ClinicalTrials.gov was selected as the data source because of its comprehensive coverage of late-stage interventional studies and standardized reporting format. Trial records were retrieved on 1 October 2025 and were examined at the trial-registration level. The review did not involve participant-level data analysis, pooled effect estimation, or comparative efficacy assessment. When posted registry results lacked sufficient quantitative detail, linked peer-reviewed publications were consulted to descriptively report effect sizes and/or p-values where available, without systematic extraction or quantitative synthesis.

### Search strategy

A structured search of *ClinicalTrials.gov* was performed to identify relevant interventional studies reporting sleep-related outcomes in PD. The search combined disease-specific and sleep-related keywords using Boolean operators to maximize retrieval sensitivity. Condition terms included *“Parkinson’s disease,” “Parkinson’s,” “Idiopathic Parkinson’s disease,” “Lewy body Parkinson’s disease,” “Primary Parkinsonism,”* and *“Shaking palsy.”* Sleep-related terms included *“sleep disturbance,” “sleep disorder,” “insomnia,” “sleep problem,” “trouble sleeping,”* and *“dyssomnia.”* These terms were applied to the “Condition” and “Other Terms” fields to ensure comprehensive retrieval.

**Fig. 1:**
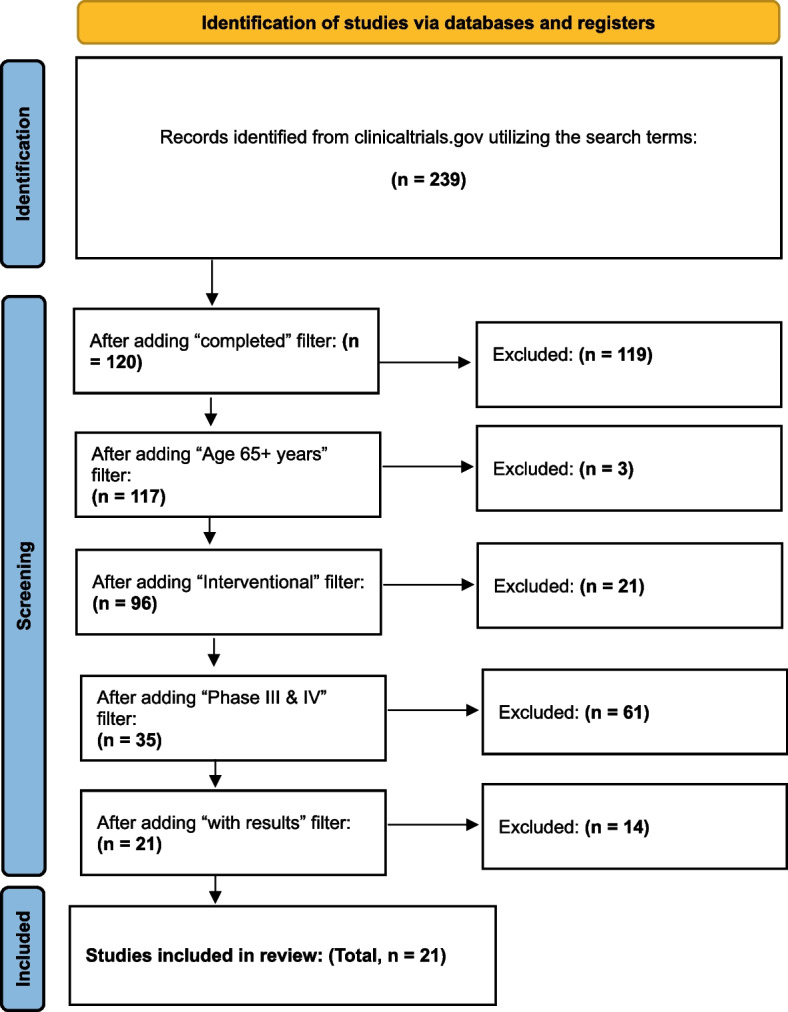
PRISMA flow diagram showing the identification and inclusion of interventional trials on the management of sleep disturbances in older adults with Parkinson’s disease (ClinicalTrials.gov, October 1, 2025)

Search results were refined using predefined registry filters to include interventional studies that were completed, had posted results, and were classified as Phase III or Phase IV. To accommodate late-stage evidence beyond the parent records, open-label extension and rollover studies were additionally screened for inclusion when explicitly linked to an eligible Phase III–IV parent trial. To ensure relevance to later life, studies were limited to those that permitted enrolment of participants aged 65 years or older, rather than requiring confirmed geriatric enrollment. Only records available in English were reviewed. Application of these criteria yielded 21 eligible trials, which formed the final dataset. The study selection process is summarized in Fig. 1 using a PRISMA-style flow diagram depicting identification, screening, and inclusion.

### Eligibility criteria

Studies were eligible for inclusion if they evaluated pharmacological, behavioral, device-based, or other interventional approaches assessing sleep-related outcomes, insomnia, circadian-related sleep dysfunction, or related non-motor symptoms in participants with PD. Eligible trials were required to report at least one sleep-related outcome as a primary, secondary, or specified exploratory endpoint. Late-stage interventional studies were operationally defined as Phase III–IV interventional trials and open-label extension/rollover studies explicitly linked to an included parent Phase III–IV trial.

To support reproducible identification and classification of sleep-related outcomes within registry records, an a priori outcome framework was applied. Sleep-related outcomes were defined as endpoints explicitly assessing sleep quality, sleep continuity, sleep architecture, circadian rhythm disturbance, or excessive daytime sleepiness using validated subjective instruments or objective sleep measures. At the screening and data-extraction stages, eligibility for “sleep-related outcomes” was operationalized using prespecified, validated instruments commonly registered in PD trials, including the Parkinson’s Disease Sleep Scale (PDSS) [30], the revised PDSS-2 [31], Scales for Outcomes in Parkinson’s Disease–Sleep (SCOPA-Sleep) [32], the Pittsburgh Sleep Quality Index (PSQI) [33], the Epworth Sleepiness Scale (ESS) [34], and the Insomnia Severity Index (ISI) [35]. Objective sleep outcomes included polysomnographic parameters (e.g., sleep efficiency, sleep latency, REM sleep metrics) and actigraphy-derived measures. When other sleep-domain instruments were specified, inclusion was determined by explicit designation as a sleep-related endpoint and alignment with the prespecified sleep constructs.

Given overlap in naming across instrument versions and the potential for misinterpretation when results are compared across non-equivalent scales, instrument handling rules were prespecified. The Unified Parkinson’s Disease Rating Scale (UPDRS) comprises four parts assessing mentation/behavior/mood (Part I), activities of daily living (Part II), motor examination (Part III), and therapy complications (Part IV) [36]. Whereas the the Movement Disorder Society–sponsored revision (MDS-UPDRS) retains a four-part structure but incorporates updated item content and scoring anchors and expanded non-motor assessment [37]; accordingly, UPDRS and MDS-UPDRS scores were not treated as directly interchangeable. Similarly, PDSS and PDSS-2 differ in response format and scoring direction (PDSS: higher scores reflect better sleep; PDSS-2: higher scores reflect greater sleep disturbance), and were not treated as interchangeable [30, 31]. Consequently, outcomes were summarized within instrument families and reported using original instrument definitions and scoring directions, without conversion or pooling across versions.

Trials were excluded if they were observational, preventive, or diagnostic in design; or if sleep-related endpoints were not specified. Studies that were withdrawn or terminated without posted results were also excluded.

### Data extraction and variables

Trial records were manually exported as a comma-separated values (CSV) file from ClinicalTrials.gov. For each eligible trial, the following variables were extracted at the trial-registration level: National Clinical Trial (NCT) identifier, study title, condition, intervention type, phase, study design, enrollment size, sponsor or collaborator, geographic location, primary and secondary outcome measures, and study start and completion dates. When multiple interventions or outcomes were reported, the principal sleep-related intervention and its corresponding sleep outcome were recorded. Each record was reviewed for internal consistency and completeness. Where registry-posted results were insufficiently detailed to characterize quantitative findings, linked peer-reviewed publications were used descriptively to report effect sizes and/or p-values when available, without systematic extraction or synthesis.

### Data analysis

The analysis was descriptive. Frequencies and proportions were calculated to summarize trial characteristics, including intervention category (pharmacologic versus non-pharmacologic), study phase, sponsorship, and sample size. Trends in intervention type, assessment tools, and geographic distribution were examined qualitatively to identify gaps in the evidence base and the representation of older adults within PD sleep research.

## Results

### Study selection

A total of 239 records were identified. After applying the completed filter, 120 records remained. Limiting to trials permitting enrollment of participants aged 65 years or older yielded 117 records (three excluded as not older-adult eligible). Restricting to interventional trials resulted in 96 records (21 excluded as non-interventional upon detailed review). Applying Phase III–IV retained 35 records (61 excluded for phase). Requiring posted results yielded 21 eligible late-stage interventional study records, all of which were included in the final analysis. The identification, screening, and inclusion process is summarised in Fig. 1.

### General characteristics of included trials

Twenty-one completed late-stage interventional studies were included in the final dataset. All trials were registered on ClinicalTrials.gov and permitted enrolment of participants aged 65 years or older with diagnosis of Parkinson’s disease. The studies collectively reflect two decades of clinical investigation into pharmacologic strategies in which sleep-related outcomes were reported in this population.

All identified trials were interventional and mostly randomized, most of which employed double-blind or active-comparator designs. Sample sizes ranged from fewer than 50 to approximately 1,000 participants, indicating that both exploratory and confirmatory approaches were represented. Most studies were multicentre and industry-sponsored.

The interventions were classified according to their principal therapeutic target rather than mechanism of action, yielding four descriptive symptom domains: insomnia and sleep maintenance, excessive daytime sleepiness and fatigue, nocturnal motor and autonomic symptoms, global sleep quality and integrated management, and neuropsychiatric or mood-associated sleep disturbances. Continuous dopaminergic stimulation (via transdermal or intestinal delivery systems) accounted for the largest proportion of trials, followed by oral dopaminergic and adjunctive pharmacologic agents.

Across studies, sleep outcomes were captured using both disease-specific and generic instruments. The Parkinson’s Disease Sleep Scale (PDSS) and Scales for Outcomes in Parkinson’s Disease–Sleep (SCOPA-Sleep) were the most frequent primary or secondary endpoints, while global measures such as the Pittsburgh Sleep Quality Index (PSQI), Epworth Sleepiness Scale (ESS), and Non-Motor Symptoms Scale (NMSS) were also commonly applied. Several studies incorporated composite or exploratory endpoints linking motor fluctuations, fatigue, or mood with subjective sleep quality.

Most trials evaluated pharmacologic agents, and no eligible non-pharmacologic, behavioral, or device-based interventions were identified. Study durations were generally short, typically 6 to 24 weeks, limiting long-term observations. Overall, the body of evidence reflects a predominance of dopaminergic and related pharmacotherapies addressing heterogeneous aspects of sleep disturbance in later-life Parkinson’s disease. A comprehensive summary of the included trials, organized by therapeutic target, is presented in Table 1.

**Table 1:** Summary of included late-stage interventional trials reporting sleep-related outcomes in Parkinson’s disease

#	NCT Number	Full Study Title	Condition / Indication	Primary and Sleep-related outcome(s)	Intervention / Protocol
A. *Insomnia and Sleep Maintenance*					
1	NCT00324896 [38]	Treatment of Insomnia in Patients With Parkinson’s Disease	Parkinson’s disease with insomnia	Change in total sleep time (TST); wake after sleep onset (WASO)	Eszopiclone & Placebo
2	NCT02729714 [39]	A Pilot Study of Suvorexant for Insomnia in Parkinson’s Disease	Parkinson’s disease with insomnia	Change in sleep efficiency measured by overnight polysomnography (PSG); Wake After Sleep Onset (WASO), Latency to Persistent Sleep (LPS)	Suvorexant & Placebo
B. *Excessive Daytime Sleepiness and Fatigue*					
3	NCT03521635 [40]	The SUSTAIN Study Compares the Effects of Sustained and Immediate-release Pramipexole on the noctUrnal Symptoms of paTients With Advanced ParkInsoN's Disease Who Also Take L-Dopa	Advanced Parkinson’s disease with nocturnal symptoms	Change from baseline in Parkinson’s Disease Sleep Scale–2 (PDSS-2) total score	Pramipexole SR & IR
4	NCT01154166 [41]	A Phase III, Randomised, Double-blind, Placebo-controlled, Parallel Group Study of Six Months Treatment With Ropinirole PR as Adjunctive Therapy in Patients With Parkinson's Disease Who Are Not Optimally Controlled on L-Dopa	Parkinson’s disease with motor fluctuations	Change in total awake time spent “off” (hours/day); Change in Parkinson’s Disease Sleep Scale (PDSS) total score	(ReQuip) Ropinirole PR & Placebo
5	NCT01397422 [42]	Extended Release Amantadine Safety and Efficacy Study in Levodopa-Induced Dyskinesia (EASED Study)	Parkinson’s disease with dyskinesia	Dyskinesia and sleep impact	ADS-5102 (extended release amantadine HCl) vs placebo
C. *Nocturnal Motor and Autonomic Symptoms*					
6	NCT01300819 [43]	Placebo-controlled Study in Patients With Parkinson's Disease to Evaluate the Effect of Rotigotine on Non-motor Symptoms	Parkinson’s disease	Total Non-Motor Symptoms Scale (NMSS) score	Rotigotine Patch
7	NCT00474058 [44]	Randomized Evaluation of the 24-Hour Coverage: Efficacy of Rotigotine	Parkinson’s disease	Change in Early Morning UPDRS Part III score; Parkinson’s Disease Sleep Scale (PDSS)	Rotigotine
8	NCT00522379 [45]	Trial to Assess Parkinson's Disease (PD) Symptom Control to Four Doses of Rotigotine in a Transdermal Patch	Parkinson’s disease	Change in daily OFF time	Rotigotine
9	NCT01227655 [46]	Efficacy and Safety of BIA 9–1067 in Idiopathic Parkinson’s Disease	Parkinson’s disease	UPDRS Part III score	BIA 9–1067 (Entacapone analog)
10	NCT01628926 [47]	A Placebo- and Ropinirole-Controlled Study for SPM 962 in Advanced Parkinson's Disease Patients	Advanced Parkinson’s disease	Change from baseline in UPDRS Part III (motor) sum score (ON state); Change from baseline in Parkinson’s Disease Sleep Scale-2 (PDSS-2) total score	(SPM 962) Rotigotine & Ropinirole & Placebo
D. *Global Sleep Quality and Integrated Management*					
11	NCT02549092 [48]	A Study to Examine the Effect of Levodopa-Carbidopa Intestinal Gel (LCIG) Therapy Relative to That of Optimized Medical Treatment (OMT) on Non-motor Symptoms (NMS) Associated With Advanced Parkinson's Disease (PD)	Advanced Parkinson’s disease	Change from baseline in modified PDSS-2 & NMSS total score; PDSS-2 total score & its domain scores (motor symptoms at night, PD symptoms at night, disturbed sleep)	LCIG continuous intestinal infusion vs optimized medical treatment (stable anti-PD and NMS medications)
12	NCT01736176 [49]	A Study to Assess the Safety and Efficacy of Levodopa-carbidopa Intestinal Gel (LCIG) for the Treatment of Non-motor Symptoms in Patients With Advanced Parkinson's Disease	Advanced Parkinson’s disease	Change from baseline in NMSS total score; NMSS sleep/fatigue domain score	LCIG & PEG-J tube procedure
13	NCT01723904 [50]	A Phase 3b, Open-Label, Safety and Efficacy Study of Rotigotine as Add-On Therapy with Low Doses of Pramipexole or Ropinirole in Patients with Advanced Parkinson's Disease	Advanced Parkinson’s disease with motor fluctuations	CGI Item 4 (side effects); Change from baseline in UPDRS II–III; absolute OFF time; PDSS-2; PSQI	Rotigotine
14	NCT03877510 [51]	Open Label Extension (OLE) Study of the Safety and Clinical Utility of IPX203 in Parkinson's Disease (PD) Participants With Motor Fluctuations	Advanced Parkinson’s disease with motor fluctuations	Number of participants with treatment-emergent adverse events (teaes); Parkinson’s Disease Sleep Scale-2 (PDSS-2): total score; change from baseline	Open-label oral IPX203 extended-release capsules for 9 months
15	NCT04380142 [52]	Study Comparing Continuous Subcutaneous Infusion Of ABBV-951 With Oral Carbidopa/Levodopa Tablets For Treatment Of Motor Fluctuations In Adult Participants with Advanced Parkinson's Disease	Advanced Parkinson’s disease with motor fluctuations	Change from baseline in average “ON” time without troublesome dyskinesia; Change from baseline in Parkinson’s Disease Sleep Scale-2 (PDSS-2) total score	Continuous subcutaneous infusion of ABBV-951 plus oral placebo vs oral carbidopa/levodopa plus placebo infusion (double-blind, double-dummy)
16	NCT03781167 [53]	A Study to Evaluate the Safety and Tolerability of ABBV-951 in Subjects with Parkinson's Disease (PD)	Parkinson’s disease	Adverse events and safety profile; Change from baseline in PDSS-2 total score	ABBV-951
17	NCT00660673 [54]	Open Label Continuation Treatment Study with Levodopa-Carbidopa Intestinal Gel in Advanced Parkinson's Disease	Advanced Parkinson’s Disease	Treatment-emergent adverse events (teaes); Number of participants with sleep attacks	LCIG & PEG-J tube procedure
18	NCT00479401 [55]	Efficacy, Safety, Tolerability of Pramipexol ER Versus Pramipexol IR Versus Placebo in Early PD Patients	Parkinson’s disease	Change from baseline in UPDRS Parts II + III total score; Change in Parkinson’s Disease Sleep Scale (PDSS)	Pramipexole ER & IR
E. *Neuropsychiatric and Comorbidity-Related Sleep Disturbances*					
19	NCT00437125 [56]	Study on the Tolerability of Duloxetine in Depressed Patients with Parkinson's Disease	Parkinson’s disease with depression	Adverse events leading to discontinuation or death; Change from baseline in Pittsburgh Sleep Quality Index (PSQI) scores	Duloxetine 30 mg once daily for 1 week, then 60 mg once daily for 11 weeks
20	NCT01568073 [57]	Efficacy and Safety of BIA 9–1067 in Idiopathic Parkinson's Disease Patients With "Wearing-off" Phenomenon	Parkinson’s disease with wearing-off phenomenon on stable L-DOPA	Change from baseline in absolute OFF-time (minutes); Change in Parkinson’s Disease Sleep Scale (PDSS) total score	BIA 9–1067 (5, 25, 50 mg) vs Entacapone vs Placebo + L-DOPA/DDCI
21	NCT02642393 [58]	Study of Urate Elevation in Parkinson’s Disease (Parkinson Study Group SURE-PD)	Parkinson’s disease	Change in MDS-UPDRS I–III total score; Change in Neuro-QOL Sleep Disturbance domain	Inosine & Placebo

*ABBV-951*, subcutaneous levodopa/carbidopa infusion, *AE* adverse event, *CGI* Clinical Global Impression, *ER* extended release, *IR* immediate release, *PR* prolonged release, *SR* sustained release, *LCIG* levodopa–carbidopa intestinal gel, *NMSS* Non-Motor Symptoms Scale, *NMS* non-motor symptoms, *PD* Parkinson’s disease, *PDSS* Parkinson’s Disease Sleep Scale, *PEG-J* percutaneous endoscopic gastrojejunostomy, *PSG* polysomnography, *UPDRS* Unified Parkinson’s Disease Rating Scale, *OMT* optimized medical treatment, *NJ* nasojejunal, *OLE* open-label extension, *HCl* hydrochloride, *OFF time* periods of reduced medication effect

### Clinical study categories and outcomes

Key findings were descriptively summarised from linked peer-reviewed publications corresponding to trials identified through ClinicalTrials.gov; when published outcome data were unavailable, results reported within the registry record were used. Registry entries were consulted to verify trial characteristics, eligibility criteria, and outcome domains. This approach was not intended as a systematic synthesis or comparative efficacy analysis.

The interventional trials were grouped according to the predominant sleep-related outcome domain reported in patients with Parkinson’s disease. Categories were defined based on the predominant symptom or outcome domain reported in the study record, including insomnia and sleep maintenance, excessive daytime sleepiness and fatigue, rapid eye movement (REM) sleep behavior disorder, circadian rhythm disruption, and mixed or non-specific sleep disturbances.

Sample sizes varied widely, ranging from small exploratory cohorts to large multicentre trials enrolling several hundred participants. The majority employed randomized designs, although study models included placebo-controlled, active-comparator, and open-label approaches. Treatment durations were heterogeneous, ranging from short-term studies of several weeks to longer-term trials extending up to nine months. Outcome measures varied between subjective sleep questionnaires and infrequently applied objective sleep measures. Adverse event reporting was available in most completed trials, with serious treatment-related events reported infrequently or not systematically captured across studies.

The following subsections (3.3.1 – 3.3.5) summarize each category of sleep disturbance and its corresponding clinical trials.

#### Insomnia and sleep maintenance

Two randomized controlled trials evaluated pharmacologic treatments for insomnia and sleep maintenance in patients with Parkinson’s disease (Table 2). A parallel-group trial of eszopiclone (*n* = 30) over 6 weeks reported improvements in nocturnal awakenings and patient-reported sleep quality compared with placebo, with a non-significant increase in total sleep time. Mild sedation was reported in 13% of participants. A double-blind crossover trial of suvorexant (*n* = 21) demonstrated improvement in insomnia severity scores, while no significant changes were observed in sleep efficiency, wake after sleep onset, or sleep latency. Reported adverse events were mild and included drowsiness and dry mouth.

**Table 2:** Summary of clinical trials investigating insomnia and sleep maintenance in Parkinson’s disease

#	Trial ID	Intervention (Comparator)	Study Design	Sample Size	Duration	Key Findings	Adverse Events
1	NCT00324896 [38]	Eszopiclone (Placebo)	Randomized, parallel-group	30	6 weeks	↑ 66 min vs 47 min (p = 0.11); fewer awakenings (p = 0.035); better sleep quality (p = 0.018)	Mild sedation (13%)
2	NCT02729714 [39]	Suvorexant (Placebo)	Randomized, double-blind crossover	21	4 weeks + 2-wk washout	Δ 8.9% vs 9.4% (p = 0.72); ISI improved (p = 0.004); no significant change in WASO or latency	Drowsiness, dry mouth (mild)

*PD* Parkinson’s disease, *y* years, *PSG* polysomnography, *ISI* Insomnia Severity Index, *WASO* wake after sleep onset, *wk* week, *min* minutes

#### Excessive daytime sleepiness and fatigue

Three randomized clinical trials examined dopaminergic and antiglutamatergic therapies in relation to excessive daytime sleepiness and fatigue in Parkinson’s disease (Table 3). A pilot phase IV study comparing sustained-release and immediate-release pramipexole (*n* = 98) reported similar improvements in sleep-related outcomes, with no significant between-group differences, while greater improvement in motor complications was observed with the sustained-release formulation. A large randomized trial of ropinirole prolonged release (*n* = 344) demonstrated significant reductions in awake “off” time and improvements in motor symptoms compared with placebo, with sleep-related outcomes reported as secondary measures. A phase III dose-finding study of extended-release amantadine (*n* = 83) showed improvements in dyskinesia-related outcomes at the 274-mg dose, although no significant effects on sleep duration were observed. Adverse events across studies were consistent with known dopaminergic side-effect profiles.

**Table 3:** Summary of clinical trials investigating excessive daytime sleepiness and fatigue in Parkinson’s disease

#	Trial ID	Intervention (Comparator)	Study Design	Sample Size	Duration	Key Findings	Adverse Events
3	NCT03521635 [40]	Pramipexole SR vs Pramipexole IR	Phase IV, multicenter, open-label, randomized, active-controlled, parallel-group (pilot)	98	18 weeks	PDSS-2 improved similarly: –13.7 (SR) vs –14.4 (IR); no significant between-group difference. SR showed greater improvement in MDS-UPDRS IV (motor complications)	Dizziness, nausea, dyskinesia, somnolence; impulse-control and hallucinations reported
4	NCT01154166 [41]	Ropinirole prolonged release (Placebo)	Randomized, double-blind, parallel-group	344	24 weeks	Reduced awake “off” time vs placebo (− 2.1 vs − 0.4 h/day; p < 0.001); improved UPDRS motor score; PDSS total score change reported	Serious AEs: 5.1% (ropinirole PR) vs 4.7% (placebo); common AEs included Dyskinesia (6%), nausea (3%), dizziness (4%), somnolence (2%)
5	NCT01397422 [42]	Amantadine ER(Go) 210 / 274 / 338 mg vs placebo	Phase III dose-finding, multicenter, randomized, double-blind, parallel-group	83	8 weeks	274 mg vs placebo: UDysRS improved (LS mean diff –11.3; p = 0.005); ON time without troublesome dyskinesia + 3.0 h; no effect on sleep duration observed (reported in the paper)	Dizziness, hallucinations most common; withdrawals due to more common at highest dose

*PD* Parkinson’s disease, *PR* prolonged release, *SR* sustained release, *IR* immediate release, *UPDRS* Unified Parkinson’s Disease Rating Scale, *MDS-UPDRS* Movement Disorder Society–Unified Parkinson’s Disease Rating Scale, *PDSS-2* Parkinson’s Disease Sleep Scale–2, *ER* extended release, *UDysRS* Unified Dyskinesia Rating Scale, *LS* least squares, *h* hours, *mg* milligrams

#### Nocturnal motor and autonomic symptoms

Five randomized controlled trials evaluated dopaminergic therapies targeting nocturnal motor and autonomic symptoms in Parkinson’s disease (Table 4). Four studies investigated transdermal rotigotine across parallel-group and dose–response designs, consistently demonstrating improvements in nocturnal symptom burden, sleep-related scales, and motor outcomes compared with placebo. Improvements were observed in PDSS-2, NMSS domain scores, and UPDRS III, with dose-dependent reductions in off-time reported in one trial. An adjunctive study of opicapone in patients receiving levodopa showed significant reductions in daily off-time relative to placebo, with sustained benefit during open-label follow-up. Across studies, adverse events were consistent with known dopaminergic effects, with application site reactions frequently reported for rotigotine.

**Table 4:** Summary of clinical trials investigating Nocturnal Motor and Autonomic Symptoms in Parkinson’s disease

#	Trial ID	Intervention (Comparator)	Study Design	Sample Size	Duration	Key Findings	Adverse Events
6	NCT01300819 [43]	Rotigotine (Placebo)	Randomized, double-blind, parallel-group	349 (283 completed)	12 weeks	Rotigotine reduced NMSS total score by 23 vs 19 for placebo (NS); greater improvement in ‘mood/apathy’ and ‘miscellaneous’ domains; motor UPDRS III improved (p = 0.002)	Nausea, somnolence, headache, application site reactions
7	NCT00474058 [44]	Rotigotine (Placebo)	Randomized, double-blind, parallel-group	287	12 weeks	Rotigotine significantly improved PDSS-2 total (− 25.9 vs − 21.9; p < 0.0001) and UPDRS III (− 27.0 vs − 23.9; p = 0.0002)	Nausea, dizziness, application site reactions
8	NCT00522379 [45]	Rotigotine 2–8 mg/24 h (Placebo)	Randomized, double-blind, dose–response	514 (409 completed)	12 weeks	Rotigotine 8 mg/24 h significantly reduced ‘off’ time by − 2.4 h vs − 1.5 h placebo; dose-dependent effect	Application site reactions, nausea, dry mouth, dyskinesia; no worsening of insomnia or somnolence
9	NCT01227655 [46]	Opicapone 25–50 mg/d (Placebo)	Randomized, double-blind, adjunct to levodopa	427	14 –15 weeks	Opicapone 50 mg/d significantly reduced mean daily off-time by 54 min vs placebo; effect maintained during 1-year open-label	Dyskinesia, constipation, dry mouth
10	NCT01628926 [47]	Rotigotine (Ropinirole / Placebo)	Randomized, double-blind, double-dummy	420	16 weeks	Rotigotine superior to placebo (− 6.4 ± 1.2 points); non-inferior to ropinirole	Application site reactions (57.7% rotigotine), otherwise no major safety concerns

*PD* Parkinson’s disease, *NMSS* Non-Motor Symptoms Scale, *UPDRS* Unified Parkinson’s Disease Rating Scale, *PDSS-2* Parkinson’s Disease Sleep Scale–2, *NS* not significant, *mg* milligrams, *h* hours, *d* day, *ON* on-medication state, off/off-time, periods of reduced medication effect

#### Global sleep quality and integrated management

Eight interventional studies evaluated global sleep quality within integrated management strategies for Parkinson’s disease (Table 5). These trials primarily examined advanced levodopa-based delivery approaches, including intestinal gel formulations and continuous subcutaneous infusion systems, as well as extended-release and adjunctive dopaminergic therapies, using randomized and open-label designs. Several studies employed randomized, controlled comparisons over short- to medium-term durations, whereas others consisted of single-arm or open-label extension phases designed to assess longer-term outcomes and safety. Study populations largely comprised patients with advanced disease, motor fluctuations, or inadequate symptom control on oral treatment. Primary and secondary outcomes included global non-motor symptom burden, sleep quality and sleep disturbance measures, motor function indices, and clinician- and patient-reported global impressions, with safety and tolerability assessed across short- and long-term follow-up periods.

**Table 5:** Summary of clinical trials investigating Global Sleep Quality and Integrated Management in Parkinson’s disease

#	Trial ID	Intervention (Comparator)	Study Design	Sample Size	Duration	Key Findings	Adverse Events
11	NCT02549092 [48]	LCIG vs Optimized Medical Treatment (OMT)	Phase 3b, open-label, randomized, parallel-group	89 (LCIG 43; OMT 44)	26 wks	NMSS total score change at Week 26: LCIG − 32.0 vs OMT − 23.8 (p = 0.410); PDSS-2 total score change: LCIG − 7.4 vs OMT − 9.0 (p = 0.509); PDSS-2 disturbed sleep domain favored LCIG (LS mean diff 1.99; p = 0.020); UPDRS II improved with LCIG (LS mean diff − 2.79; p = 0.006); CGI-C favored LCIG (p < 0.001)	Serious AEs: 20.9% LCIG vs 9.1% OMT; common AEs included stoma-site complications, abdominal pain, dyskinesia, falls, insomnia, hallucinations; no treatment-period deaths
12	NCT01736176 [49]	Levodopa–carbidopa intestinal gel (LCIG) (none)	Open-label, single-arm, multicenter, Phase 3	39	60 wks	NMSS total score improved at Week 12 (LS mean − 17.6 ± 3.6; p < 0.001) and Week 60 (− 11.8 ± 3.95; p = 0.004); NMSS sleep/fatigue domain improved at Week 12 and Week 60	Serious AEs: 20.5%; common AEs included GI disorders, stoma-site complications, falls, anxiety, insomnia, orthostatic hypotension
13	NCT01723904 [50]	Rotigotine patch add-on to low-dose oral DA	Phase 3b, open-label, single-arm	90 (80 completed)	8 wks	CGI Item 4 (side effects: 83/89 (93.3%) scored 1–2; 6/89 (6.7%) scored 3–4. UPDRS III (ON): − 5.3 ± 8.3 points. Sleep outcomes: PDSS-2 − 3.2 ± 7.5 points; PSQI − 0.7 ± 3.0 points	Serious AEs: 5.56% (5/90); Any AE: 38.9% (35/90); Common AEs ≥ 7%: application-site pruritus 13.3%, dizziness 10.0%, orthostatic hypotension 10.0%, nausea 7.8%, dyskinesia 7.8%
14	NCT03877510 [51]	IPX203 (single-arm; no comparator)	Phase 3, Open-label, multicentre	419 (352 completed)	9 months	Change from baseline to Month 9: MDS-UPDRS total + 2.7 ± 16.44 (n = 391); PDSS-2 total − 0.1 ± 8.92 (n = 386)	Any TEAE 52.7% (221/419); serious AEs 10.0% (42/419); mortality 1.43% (6/419)
15	NCT04380142 [52]	ABBV-951 (foslevodopa/foscarbidopa) CSCI vs oral LD/CD	Phase 3, randomized, double-blind, active-controlled	174 randomized (ABBV-951 n = 74; LD/CD n = 67 analyzed)	12 wks	ON time without troublesome dyskinesia increased more with ABBV-951 than LD/CD (+ 2.72 vs + 0.97 h; p = 0.008). OFF time reduced (− 2.75 vs − 0.96 h; p = 0.005). Early morning OFF: 17.0% vs 63.3% (p ≤ 0.001). PDSS-2 sleep score improved (− 7.92 vs − 2.52; p ≤ 0.001)	Serious AEs: 8.1% (ABBV-951) vs 6.0% (LD/CD). Infusion-site reactions common with ABBV-951 (erythema 27.0%, pain 25.7%). One death in LD/CD group; none with ABBV-951
16	NCT03781167 [53]	ABBV-951 (foslevodopa/foscarbidopa) CSCI – open-label extension	Open-label, single-arm, Phase 3	244 (173 completed)	Up to 52 wks	Daily OFF time decreased from baseline; ON time without troublesome dyskinesia increased. PDSS-2 total score improved, indicating reduced sleep disturbance. Improvements observed from Week 6 and sustained through Week 52	Any TEAE: 218/244 (89.3%); serious AEs: 63/244 (25.8%); infusion-site reactions ≥ grade D/ ≥ 5: 25/244 (10.2%); all-cause mortality: 5/244 (2.05%)
17	NCT00660673 [54]	LCIG via PEG-J (long-term open-label)	Phase 3, multinational	262	Mean 4.1 yrs	Mean daily “off” time − 3.97 h from initial LCIG infusion (*p* < 0.001); “on” time without troublesome dyskinesia + 3.86 h (*p* < 0.001)	Serious AEs: 60.7%; mortality: 22.5%; device/procedure complications common; insomnia and sleep attacks reported
18	NCT00479401 [55]	Pramipexole ER vs Pramipexole IR vs Placebo	Randomized; parallel assignment; triple-masked (participant/investigator/outcome assessor); treatment purpose	539	33 weeks	UPDRS II + III improved with pramipexole ER (− 8.6) and IR (− 8.8) vs placebo (− 3.8); ER non-inferior to IR. PDSS scores increased modestly across groups	Serious AEs: 7.2% (ER), 5.2% (IR), 3.9% (placebo). Common AEs: somnolence, nausea, dizziness, constipation

*PD* Parkinson’s disease, *LCIG* levodopa–carbidopa intestinal gel, *OMT* optimized medical treatment, *NMSS* Non-Motor Symptoms Scale, *PDSS-2* Parkinson’s Disease Sleep Scale–2, *UPDRS* Unified Parkinson’s Disease Rating Scale, *CGI-C* Clinical Global Impression of Change, *PGIC* Patient Global Impression of Change, *QoL* quality of life, *AE* adverse event, *SAE* serious adverse event, *DA* dopamine agonist, *LD* levodopa, *CD* carbidopa, *LD/CD* levodopa/carbidopa, *ER* extended release, *IR* immediate release, *CSCI* continuous subcutaneous infusion, *TEAE* treatment-emergent adverse event, *PEG-J* percutaneous endoscopic gastrojejunostomy, *UTI* urinary tract infection, *LS* least squares, *CI* confidence interval, *ON* on-medication state, *Off* off-medication state, *h* hours, *wks* weeks, *mos* months, *yrs* years, *y* years

#### Neuropsychiatric and comorbidity-related sleep disturbances

Three interventional studies investigated sleep disturbances associated with neuropsychiatric symptoms and comorbid conditions in Parkinson’s disease (Table 6). These trials examined pharmacologic interventions targeting depression, non-motor symptom burden, and disease-modifying pathways, using both randomized controlled and open-label designs. Study populations included patients with Parkinson’s disease and comorbid depression, non-motor symptom manifestations, or early-stage disease with specific biochemical profiles. Outcomes assessed encompassed sleep quality measures, neuropsychiatric symptom scales, quality-of-life indices, and global clinical assessments, alongside motor and functional outcomes and systematic safety monitoring.

**Table 6:** Summary of clinical trials investigating neuropsychiatric and comorbidity-related sleep disturbances in Parkinson’s disease

#	Trial ID	Intervention (Comparator)	Study Design	Sample Size	Population	Duration	Key Findings	Adverse Events
19	NCT00437125 [56]	Duloxetine 30–60 mg QD	Single-group, open-label	151	PD patients with depression	12 weeks	Duloxetine improved sleep quality (PSQI − 3.2), HAMD-17 (− 10.1), BDI (− 12.0), and PDQ-39 (− 7.7); small changes in UPDRS II/III	Nausea, insomnia, headache, somnolence; serious AEs in 3/151 participants
20	NCT01568073 [57]	BIA 9–1067 (Entacapone analog)	Randomized, double-blind, placebo-controlled	600	Idiopathic PD with non-motor symptoms	14–15 weeks	Primary: change in absolute OFF-time (min, mean ± SE): placebo − 56.0; entacapone − 96.3; opicapone 5 mg − 91.3; 25 mg − 85.9; 50 mg − 116.8. Secondary: PDSS mean scores increased from baseline to endpoint across all treatment arms	Serious AEs (%): placebo 4.96; entacapone 6.56; opicapone 5 mg 3.28; 25 mg 0.84; 50 mg 3.48. Non-serious AEs reported across groups, including dyskinesia, insomnia, somnolence, dizziness, and gastrointestinal events
21	NCT02642393 [58]	Inosine (Placebo)	Randomized, double-blind, placebo-controlled	298	Early PD with serum urate < 5.8 mg/dL	2 years	Primary: change in MDS-UPDRS I–III total score (inosine 11.116 vs placebo 9.860 points/year; p = 0.183). Secondary outcomes included Neuro-QOL, PDQ-39, MoCA, Schwab & England ADL, and disability requiring dopaminergic therapy	Serious AEs: 10.88% (inosine) vs 14.09% (placebo). Nephrolithiasis: inosine 4.08% (serious), 8.16% (non-serious) vs placebo 0–2.68%. Mortality: 1 inosine, 0 placebo

*PD* Parkinson’s disease, *QD* once daily, *PSQI* Pittsburgh Sleep Quality Index, *HAMD-17* 17-item Hamilton Depression Rating Scale, *BDI* Beck Depression Inventory, *PDQ-39* Parkinson’s Disease Questionnaire-39, *UPDRS* Unified Parkinson’s Disease Rating Scale, *NMSS* Non-Motor Symptoms Scale, *AE* adverse event, *mg* milligrams, *dL* deciliter

### Sleep outcomes and assessment tools

The included trials used a diverse range of instruments to evaluate sleep and related non-motor outcomes (Tables 2, 3, 4, 5 and 6). The Parkinson’s Disease Sleep Scale (PDSS and PDSS-2) and the Scales for Outcomes in Parkinson’s Disease–Sleep (SCOPA-Sleep) were the most frequently applied measures, serving as either primary or secondary endpoints in more than half of the studies. These disease-specific instruments captured key aspects of nocturnal sleep quality, sleep fragmentation, and early-morning motor symptoms that are particularly relevant to Parkinson’s disease.

Generic sleep and alertness questionnaires, including the Pittsburgh Sleep Quality Index (PSQI), Epworth Sleepiness Scale (ESS), Insomnia Severity Index (ISI), and Fatigue Severity Scale (FSS), were used variably to complement PD-specific tools. Broader non-motor or global outcome scales, such as the Non-Motor Symptoms Scale (NMSS), Clinical Global Impression (CGI), and Patient Global Impression of Change (PGIC), were also reported to link sleep improvement with overall quality of life. Objective measurements were uncommon. Only two studies incorporated polysomnography or actigraphy to quantify parameters such as total sleep time, sleep efficiency, and wake-after-sleep-onset. Most trials relied exclusively on self-reported data, limiting comparability and sensitivity to physiological changes. Follow-up durations were generally short, ranging from six to twenty-four weeks, constraining evaluation of long-term outcomes or circadian adaptation. Overall, the assessment landscape demonstrated considerable heterogeneity and a predominant reliance on subjective rather than multimodal approaches. These findings are contextualized in the efficacy and safety results presented in Sect. Reported efficacy and safety profiles.

### Reported efficacy and safety profiles

Across the included late-phase clinical trials, sleep-related efficacy was generally reported as modest and variable across intervention classes, with the clearest patterns emerging within each trial category as summarized in Tables 2, 3, 4, 5 and 6. Insomnia-targeted studies (NCT00324896 [38], NCT02729714 [39]) reported limited separation on pre-specified sleep endpoints, with improvement signals concentrated in selected secondary sleep measures (Table 2). Trials primarily designed around motor fluctuation or non-motor symptom outcomes (NCT01154166 [41], NCT03521635 [40], NCT01397422 [42]) reported sleep-domain changes as part of broader clinical response patterns rather than as dominant endpoints (Table 3). In advanced-disease and integrated management programs, improvements were most consistently described within global non-motor, quality-of-life, and nocturnal symptom domains (e.g., NCT02549092 [48], NCT01736176 [49], NCT04380142 [52], NCT03781167 [53]) (Table 5).

When considered by category, the insomnia trials suggested that symptom-level sleep gains can occur without robust between-group effects on pre-specified endpoints in small late-phase cohorts (Table 2). Within dopaminergic and adjunctive pharmacotherapy trials, the direction of benefit most commonly aligned with better nocturnal motor control, reduced “off” burden, or reduced non-motor symptom burden, with sleep scales improving in some studies but not consistently distinguishing between formulations or comparators (Tables 3, 4 and 5). Rotigotine programs evaluating nocturnal/early-morning dysfunction (e.g., NCT00474058 [44], NCT01300819 [43], NCT00522379 [45]) generally reported favorable changes in sleep-related domains when included, alongside motor improvements (Table 4). For neuropsychiatric or comorbidity-linked sleep disturbance, duloxetine (NCT00437125 [56]) showed improvement in sleep quality within a broader mood response profile, whereas inosine (NCT02642393 [58]) did not demonstrate benefit on the primary clinical decline endpoint despite achieving expected biological effects (Table 6).

Safety profiles were consistent with the intervention type and route of administration across Tables 2, 3, 4, 5 and 6. Sedation-related effects predominated in insomnia pharmacotherapy (Table 2). Dopaminergic and adjunctive trials commonly reported expected events such as somnolence, nausea, dizziness, dyskinesia, and neuropsychiatric symptoms in susceptible participants (Tables 3 and 4). For continuous delivery and device-based approaches (LCIG and subcutaneous infusion; NCT01736176 [49], NCT00660673 [54], NCT04380142 [52], NCT03781167 [53]), adverse event burden was largely driven by procedure/device or infusion-site complications, with falls and infections also reported in advanced cohorts (Table 5). Overall, serious adverse events were reported across several programs, but patterns were dominated by expected class effects and route-specific complications rather than new safety signals (Tables 2, 4, 5 and 6).

## Discussion

### Overview of main findings

This registry-based analysis identified twenty-one completed Phase III and Phase IV interventional trials evaluating sleep-related outcomes in older adults with Parkinson’s disease. The evidence base was small and largely restricted to pharmacological, dopaminergic strategies; no completed behavioural, cognitive or device-based interventions met inclusion criteria. Most studies were short term and relied on subjective instruments such as the Parkinson’s Disease Sleep Scale (PDSS/PDSS-2) and the Scales for Outcomes in Parkinson’s Disease–Sleep (SCOPA-Sleep), whereas objective assessments, including polysomnography, were rarely used. Across interventions, efficacy was modest and primarily reflected improvements in patient-reported sleep quality and non-motor domains. Continuous dopaminergic delivery systems, including levodopa–carbidopa intestinal gel and subcutaneous levodopa/carbidopa infusion, produced the most consistent gains, and adverse events were generally mild to moderate and aligned with known pharmacological profiles. Collectively, these findings indicated incremental rather than transformative benefits and highlighted persistent gaps, particularly the under-representation of frail older adults and the absence of long-term, multimodal designs. These observations provided the basis for contextual interpretation and the identification of future research priorities.

### Comparative interpretation with previous literature

#### Dopaminergic and adjunctive therapies

The predominance of dopaminergic approaches reflected the historical therapeutic framework of Parkinson’s disease. Agents such as rotigotine, pramipexole and levodopa–carbidopa intestinal gel yielded modest but reproducible improvements in subjective sleep quality and overall non-motor burden, consistent with evidence that continuous dopaminergic stimulation mitigates nocturnal akinesia and early-morning “off” periods, thereby improving perceived rest and morning function [59, 60]. Typical mean PDSS or NMSS improvements remained within a 3–5 point range, below conventional thresholds of clinical relevance. Objective verification of restored sleep architecture was seldom achieved [16, 61]. Tolerability issues such as excessive daytime sleepiness, vivid dreaming and orthostatic hypotension persisted, particularly among older adults with autonomic vulnerability. Adjunctive agents targeting adenosine and orexin pathways produced heterogeneous results, underscoring that pharmacological modulation alone addresses only part of the disrupted sleep–wake continuum.

#### Non-dopaminergic and behavioural interventions

The absence of behavioural and circadian-based interventions among completed late-phase trials exposed a significant translational gap. Outside this registry, smaller studies have suggested that cognitive-behavioural therapy for insomnia, structured physical activity and bright-light therapy can improve sleep quality, circadian alignment and mood in Parkinson’s disease with minimal risk [21–23]. These modalities target mechanisms such as maladaptive cognitions, deconditioning and circadian misalignment that are not modified by dopaminergic therapy. Taken together, current and prior evidence indicates that durable, multidimensional improvement will require integration of pharmacological and behavioural strategies.

### Methodological strengths and limitations of included studies

Methodological strengths included frequent randomised, double-blind designs, multicentre recruitment and use of validated disease-specific questionnaires (PDSS-2, SCOPA-Sleep), which enhanced internal validity and clinical relevance [61]. Several trials used active comparators and applied consistent safety monitoring. However, follow-up durations were relatively short (6–24 weeks), limiting assessment of sustained benefit or longer-term outcomes. Objective measures such as polysomnography and actigraphy were seldom deployed, restricting independent verification of questionnaire-based improvements [25, 62]. Frail and cognitively impaired adults were likely under-represented due to restrictive eligibility criteria, limiting generalisability to real-world geriatric populations [26, 27]. Outcome heterogeneity, limited standardisation of adverse-event reporting and incomplete registry reporting further constrained cross-trial comparability. In addition, selective reporting of sleep outcomes and potential publication bias, including under-registration of negative studies, may have inflated apparent efficacy. Future research should employ longer-term, multimodal trial designs incorporating objective metrics, geriatric stratification and harmonised endpoints to enhance generalisability.

### Geriatric and frailty considerations in sleep research

Older adults with Parkinson’s disease exhibit substantial heterogeneity in frailty, multimorbidity, and cognitive reserve, each influencing sleep physiology and therapeutic responsiveness. Restrictive eligibility criteria have excluded these groups from most clinical trials, reducing real-world applicability [26, 27]. Frailty complicates both presentation and interpretation, as fatigue and slowed gait may overlap with motor symptoms, leading to diagnostic uncertainty and underestimation of vulnerability [63, 64]. Reduced circadian resilience further predisposes to fragmented sleep, nocturnal confusion, and excessive daytime sleepiness, particularly in those with advanced disease and multimorbidity [65]. Incorporating validated frailty indices such as the Fried phenotype or the Clinical Frailty Scale would enable stratified analyses across vulnerability levels and facilitate the identification of therapeutic responsiveness within heterogeneous geriatric cohorts [66]. Pragmatic designs accommodating multimorbidity, cognitive impairment, and caregiver participation [67, 68], together with interdisciplinary models integrating neurology, geriatrics, and sleep medicine, are essential to optimise treatment sequencing for functionally vulnerable populations. Advances in chronobiology and dopaminergic sleep regulation further underscore the importance of multimodal, frailty-informed approaches for improving sleep health in older adults with Parkinson’s disease [69, 70].

### Methodological advances and future directions in sleep measurement

Progress will depend on adoption of standardised, multimodal assessment frameworks that integrate subjective perception with physiological validation. Traditional reliance on PDSS, PSQI and SCOPA-Sleep offers practicality but remains susceptible to recall bias and cognitive variability [71, 72]. Evidence that neurodegenerative phenotypes exhibit distinct sleep spindle and REM signatures supports inclusion of objective modalities [73]. Combining actigraphy and polysomnography with validated questionnaires would strengthen construct validity and enable mapping across disease stages. Emerging home-based sensors permit longitudinal, ecologically valid monitoring but require validation for usability and data privacy in frail cohorts [74]. Standardised data-sharing frameworks and common outcome sets would facilitate meta-analytic synthesis, while machine-learning approaches may identify subtle biomarkers predictive of progression [75].

### Clinical and public-health implications

Pharmacological optimisation remains necessary for nocturnal motor control but is insufficient to address the multifactorial nature of poor sleep in Parkinson’s disease. A geriatric-centred model that integrates cognitive-behavioural therapy for insomnia, structured exercise and light-based interventions alongside medication management is supported by emerging evidence [21–23]. Multidisciplinary programmes involving neurology, geriatrics, sleep medicine and rehabilitation could improve sleep, reduce daytime somnolence and lessen caregiver burden. From a public-health perspective, optimising sleep may mitigate falls, preserve cognition and reduce healthcare utilisation. Policy efforts that promote inclusive recruitment of older and frail adults are critical to generate equitable evidence [26].

## Conclusion

This registry-based synthesis demonstrated that dopaminergic and related pharmacological interventions produce modest improvements in subjective sleep outcomes, whereas sustained and comprehensive management remains limited. Behavioural and circadian-based therapies show promise but are under-represented in later-phase trials. Future research should adopt longitudinal, multimodal approaches that incorporate objective sleep measures and frailty stratification to ensure applicability to those most affected. Improving sleep in Parkinson’s disease is a quality-of-life imperative for ageing individuals and is best addressed through an integrated, multidisciplinary framework.

## Data Availability

The datasets generated and/or analyzed during the current study are available in the Clinicaltrials.gov repository.

## Abbreviations

ABBV-951: Continuous subcutaneous levodopa/carbidopa infusion
AE: Adverse event
BDI: Beck Depression Inventory
CBT-I: Cognitive-behavioral therapy for insomnia
CGI: Clinical Global Impression
CGI-C: Clinical Global Impression–Change
CI: Confidence interval
CSCI: Continuous subcutaneous infusion
DA: Dopamine agonist
EDS: Excessive daytime sleepiness
ER: Extended release
ESS: Epworth Sleepiness Scale
FSS: Fatigue Severity Scale
HAMD-17: 17-Item Hamilton Depression Rating Scale
HCl: Hydrochloride
IR: Immediate release
ISI: Insomnia Severity Index
LCIG: Levodopa–carbidopa intestinal gel
LD/CD: Levodopa/carbidopa
LS: Least squares
MDS-UPDRS: Movement Disorder Society–Unified Parkinson’s Disease Rating Scale
NMSS: Non-Motor Symptoms Scale
NMS: Non-motor symptoms
NJ: Nasojejunal
OLE: Open-label extension
OMT: Optimized medical treatment
PD: Parkinson’s disease
PDQ-39: Parkinson’s Disease Questionnaire–39
PDSS / PDSS-2: Parkinson’s Disease Sleep Scale / Parkinson’s Disease Sleep Scale–2
PEG-J: Percutaneous endoscopic gastrojejunostomy
PGIC: Patient Global Impression of Change
PR: Prolonged release
PSG: Polysomnography
PSQI: Pittsburgh Sleep Quality Index
QoL: Quality of life
RBD: REM sleep behavior disorder
REM: Rapid eye movement
SAE: Serious adverse event
SCOPA-Sleep: Scales for Outcomes in Parkinson’s Disease–Sleep
SR: Sustained release
TEAE: Treatment-emergent adverse event
UDysRS: Unified Dyskinesia Rating Scale
UPDRS: Unified Parkinson’s Disease Rating Scale
WASO: Wake after sleep onset
ON/Off: On-medication / off-medication state
